# Monitoring ^60^Co activity for the characterization of the sorption process of Co^2+^ ions in municipal activated sludge

**DOI:** 10.1007/s10967-013-2821-3

**Published:** 2013-11-09

**Authors:** Vladimír Frišták, Martin Pipíška, Michaela Valovčiaková, Juraj Lesný, Marián Rozložník

**Affiliations:** Department of Ecochemistry and Radioecology, University of SS. Cyril and Methodius, J. Herdu 2, Trnava, 917 01 Slovak Republic

**Keywords:** Dried activated sludge, Co^2+^, Sorption, FT-IR, SEM-EDX

## Abstract

In large volumes produced activated sludges from wastewater treatment plants (WWTPs) with low concentrations of heavy metals can be utilized as agricultural fertilizers and soil conditioners. Increased contents of toxic xenobiotics are limiting factors that affect the utilization of these heterogeneous wastes. The main aim of our paper was to show the utilization of dried activated sludge (DAS) from municipal WWTP as potential Co^2+^ ions sorbent i.e. for non-agricultural purposes. The radio indicator method by radionuclide ^60^Co and γ-spectrometry for characterization DAS sorption properties was used. DAS soluble and solid fractions were characterized by biochemical, ETAAS and CEC analysis. The sorption of Co^2+^ ions by DAS was rapid process and equilibrium was reached within 2 h. Sorption capacity of DAS (*Q*) increased with the initial concentration of CoCl_2_ in the range from 100 to 4,000 μmol l^−1^, reaching 20 and 160 μmol g^−1^. Obtained *Q* values were depent on pH value from 2.0 to 8.0. The maximum sorption capacity (*Q*_max_) of DAS at pH 6 calculated from mathematical model of Langmuir adsorption isotherm was 175 ± 9 μmol g^−1^. FT-IR analyses showed the crucial role of carboxyl functional groups of DAS surfaces on cobalt uptake. For confirmation ion-exchange mechanism in sorption process of Co^2+^ ions by DAS scanning electron microscopy and EDX analysis were used.

## Introduction

The radionuclide ^60^Co is present in liquid wastes released from the nuclear power reactors, and is also widely used in research applications. The adsorption of radiocobalt by clay and organic materials changes the physico-chemical form of nuclide and controls the diffusion and migration of cobalt in waters and environment [1]. Organic pollutants can be decomposed but inorganic as soon as toxic and radiotoxic metals are redistributed in different parts of biosphere. Incorporation of nuclide into biomass can be presented by biosorption or bioaccumulation depending on biological activity. Microbial community of bacteria isolated from nuclear fuel pools are able to bioaccumulate ^60^Co with high efficiency [2]. ^60^Co as radiotracer can be also effective used in study of cobalt sorption mechanism by variable types of sorbents from the point of the impact of radioactive and toxic waste disposal on the environment. Biological materials have effective sorption capacity and cheaper regeneration cost as compared to conventional inorganic sorbents and ion-exchange resins [3]. Processes used in conventional treatment of waste water routinely such as redox transformation, ion exchange, filtration, osmosis, and precipitation have several limitations in comparison with biosorbent alternative methods [4]. Biosorbents are potential inexpensive and regenerable materials for removal heavy metals from liquid wastes. Similarly are able to preconcentrate the elements from high dilute solutions. Bivalent metal ions such as Co^2+^ can be removed from aqueous solutions not only by inorganic sorbents, such as zeolites [5] but also by sorbents prepared from microbial biomass [6]. Increased concentrations of sewage sludges from wastewater treatment plants are alarming in environmental protection. Sludges with low toxic metals concentration can be used as fertilizer and soil conditioner in agriculture. Heterogeneity and instability of vital sludge biomass can limit these applications. On the other hand pressure of industrialization and urbanization needs suitable solution of sludges problem. Utilization of sewage sludges in non-agricultural application such as heavy metal sorbents is one of the economically acceptable ways of its remediation recovery. Remediation applications of sludges were extensive described in wide range of scientific papers [7–9]. Activated sludge to its composition offers ability for biosorption or bioleaching of the bivalent metal ions from wastewater. Sludge is composed of both live and dead microbial mass. Adsorption, ion exchange and precipitation on sorbent surface are predominant in the process of metal uptake. Only small fraction of Me^2+^ ions is localized intracellularly. Utilization of dead microbial cells has advantage because of toxic metal ions do not affect on biological processes of system. Biomass of activated sludge is rich material in concentration of humic substances. These substances may influence the sorption of cobalt ions onto surface of sludge components through their ability to complex Me^2+^ ions and their potential involvement in redox reactions. Dried activated sludge (DAS) as an effective biomaterial in sorption processes contains different functional groups such as carboxylic acid, carboxyl and amine groups. Many reports have shown that aldehyde, carboxyl, sulfhydryl, phosphoryl, hydroxyl, amine groups of sorbents play a crucial role in elimination of metal ions from aqueous solutions [4, 10, 11]. Treatment and modification methods such as heating, freezing, acido-basic treatment, autoclaving have the main effect to functional groups surface formation [12, 13]. Sorbent pretreatment can cause the solubilisation and release of sludge polymers such as polysaccharides and proteins, which have a high affinity to heavy metal ions [14]. Acidic functional groups of biopolymers play a crucial role in Co^2+^ sorption mechanism by sewage sludge [15]. Their formation depends on many operational parameters such as pH, initial concentration of CoCl_2_ in reaction solution, temperature, contact time and present of competitive ions (co-ions). These sorption effects were well described by Marešová et al. [16]. Our previous paper [8] confirmed the usability of well-known adsorption isotherms by Langmuir and Freundlich for fitting and description equilibrium data of Co^2+^ ions sorption by dried industrial activated sludge.

Based on the results mentioned above, mechanism of Co^2+^ ions uptake by municipal activated sludge should be sufficiently conclusive. The main aim of the present paper was to investigate sorption of Co^2+^ ions by DAS using radio indicator methods by ^60^Co. Effects of contact time, initial concentration of CoCl_2_ in solution and pH on cobalt sorption process was studied. Adsorption models of Langmuir and Freundlich isotherms were applied to obtain sorption parameters. Mechanism of sorption process was studied by FT-IR and SEM analyses.

## Experimental

### Dried activated sludge

For sorption experiments suspension of activated sludge (18 g l^−1^ dw, pH 6.95) from mechanical to biological wastewater treatment plant (Zeleneč, Slovak Republic) was used. Obtained sludge samples were oven dried at 105 °C, ground and sieved. After homogenization of DAS, fraction <450 μm was used in sorption experiments.

### Solutions and reagents

For sorption experiments standardized solution ^60^CoCl_2_ (0.02 g l^−1^ CoCl_2_ in 3 g l^−1^ HCl, Czech Institute of Metrology, Czech Republic (CR) was used. All chemicals used in experiments were of analytical reagent grade. The solutions were prepared using ultrapure water with conductivity <0.05 μS cm^−1^.

### Determination of cation exchange capacity (BaCl_2_ method)

To determine the cation exchange capacity (CEC) of the sludge sorbent modificated method with BaCl_2_ [17] was used. DAS was suspended in 0.1 mol l^−1^ BaCl_2_ (83.3 g l^−1^) and mixed on a laboratory shaker for 1 h at 22 °C at 150 rpm. Phases were separated by centrifugation (5 min at 5,000 rpm). This procedure was repeated twice. For next step of CEC determination, sludge sediment was resuspended in 3 ml of 0.025 mol l^−1^ BaCl_2_ and agitated for 19 h at 22 °C. After separation of phases 3 ml of 0.02 mol l^−1^ MgSO_4_ was added to sludge sediment. After next agitation (19 h at 22 °C) and phases separation CEC value by chelatometric determination of Mg^2+^ ions in 0.02 mol l^−1^ Na_2_EDTA standardized liquid phase was determined. The CEC was calculated according equation:

1 $$ CEC = \frac{{({M_0}{V_0} - M{V_v})\;\varepsilon }}{{{{10}^{ - 3}}}} $$

where *CEC* is cation exchange capacity (meq 100 g^−1^), *M*_0_ is molar concentration of magnesium added to the sample (mol l^−1^), *V*_0_ is volume of solution of magnesium added to the sample (l), *M* is molar concentration of the magnesium in the leachate (mol l^−1^), *V*_*v*_ is volume of obtained extract (l) and *ε* is the conversion factor that has for the bivalent ions and amount 0.25 g of sorbent value 800 meq (100 g mol^−1^).

### Sorption experiments of Co^2+^ ions by DAS

#### Effect of Co^2+^ ions initial concentration

Sorption experiments were carried out by suspending 0.02 g of DAS in 8 ml of reaction solution with initial concentration of CoCl_2_ in the range from 100 to 4,000 μmol l^−1^ spiked with 15 μl radioindicator ^60^CoCl_2_ (63 kBq l^−1^). The sorption was realized at pH 6 and temperature 22 °C. After agitation on a rotary shaker (4 h, 40 min^−1^, 22 °C), sediment of DAS was separated. Sediment was washed twice by ultrapure water, and radioactivity of sediment and liquid phase was measured. Sorption of cobalt ions was calculated according to:

2 $$ {Q_{eq}} = \frac{{({C_0} - {C_{eq}}) \times V}}{m} $$

where *Q* is the uptake (μmol g^−1^), *C*_0_ and *C*_*eq*_ are the liquid-phase concentrations of metal at initial and equilibrium (μmol l^−1^), *V* is the volume (l), and *m* is the amount of dried biosorbent (given in grams). The obtained data were evaluated by mathematical models of adsorption isotherms under terms of Langmuir and Freundlich (Table 1). Maximum sorption capacity of DAS was calculated by non-linear regression using the program MicroCal Origin 8.0 Professional (OriginLab Corporation, Northampton, USA).

**Table 1:** Models of adsorption isotherms used to describe the sorption equilibrium of single metal system

Adsorption model	Equation	Coefficients
Langmuir	${Q_{eq}} = \frac{{b{Q_{\hbox{max} }}{C_{eq}}}}{{1\,+ \, b{C_{eq}}}}$	*Q*_*eq*_ amount of sorbed metal at equilibrium, *b* isotherm coefficient characterizing affinity sorbent to metal ion in solution, *Q*_*max*_ maximum metal sorption capacity at saturated sorbent binding sites, *C*_*eq*_ metal equilibrium concentration in solution
Freundlich	${Q_{eq}} = K{C_{eq}}^{(1/n)}$	*Q*_*eq*_ amount of sorbed metal at equilibrium, *K,n* Freundlich empirical constants characterizing parameters of sorption process, *C*_*eq*_ metal equilibrium concentration in solution

#### Effect of reaction time

Kinetics study was performed with batch sorption experiments of Co^2+^ ions by DAS (2.5 g l^−1^) at reaction time in the range from 5 to 1,440 min. DAS biomass was resuspended in 8 ml of 1,000 μmol l^−1^ CoCl_2_ reaction solution spiked with 15 μl radio indicator ^60^CoCl_2_ (63 kBq l^−1^). The sorption system was shaken on a rotary shaker (40 min^−1^, 22 °C) at pH 6. In the end of experiment sediment of DAS was washed twice by ultrapure water, and radioactivity of both sediment and liquid phase was measured. Sorption of cobalt ions was calculated according to Eq. 2.

#### Effect of reaction pH

Dry matter of activated sludge (2.5 g l^−1^) was resuspended in 8 ml of 1,000 μmol l^−1^ CoCl_2_ reaction solution spiked with 15 μl radioindicator ^60^CoCl_2_ (63 kBq l^−1^). The sorption system was shaken on a rotary shaker (40 min^−1^, 22 °C) at pH in the range from 2 to 8. After agitation and centrifugation of reaction system sediment of DAS was washed twice by ultrapure water, and radioactivity of both sediment and liquid phase was measured. Sorption of cobalt ions was calculated according to Eq. 2 as above.

#### Radiometric analysis

For the analysis and measurement of ^60^Co radioactivity in DAS sediment and supernatants scintillation gamma detector 54BP54/2-X 76BP76/3 with NaI(Tl) crystal (Scionix, The Netherland (NL) was used. Experimental data were evaluated by software Scintivision 32 (Ortec, USA). The energy of γ-photons was 1,173.24 keV.

#### GFAAS analysis

The elemental content of cobalt was measured in representative samples of DAS by atomic absorption spectrometry with graphite furnace (GFAAS) device Shimadzu AA-6300 (USA) with electrothermal atomizers Shimadzu GFA-EX7i using an automatic dispenser Shimadzu ASC 6100 and background correction method of Smith-Hieftje, after microwave digestion of the samples MW system Multiwave 3000 (Anton Paar GmbH, Australia).

#### FT-IR analysis and SEM-EDX analyses

Spectral analysis of DAS in infrared region was used to determine binding characteristics and the determination of functional groups of sludge. The surface functional groups of DAS were detected by infrared spectroscope with Fourier transformation (FT-IR) (Nicolet NEXUS 470, Thermo Scientific, USA). The spectra were recorded from 4,000 to 400 cm^−1^. The intra-structures of DAS sorbent and surface structure analysis of Ca pretreated sludge before and after sorption of Co^2+^ ions were obtained by scanning electron microscopy (SEM) using electron microscope VEGA 2 SEM (TESCAN s.r.o., Czech Republic) and EDX microanalysis by QUANTAX QX2 detector (RONTEC, Germany) for electron dispersive X-ray analysis. The analyses was performed at voltage 30 kV, vacuum pressure 9.0 × 10^−3^ Pa and magnification 250×.

## Results and discussion

### Physico-chemical characteristic of DAS

We found out that sludge from the aerobic phase of municipal WWTP had a well-type sedimentation form in aqueous reaction system. To determine cation exchange capacity (CEC) the known relationship (Eq. 2) was used. CEC of DAS was 15 meq 100 g^−1^. Our previous papers [8, 15] showed that activated sludges from industrial WWTP had a comparable CEC values determined by the same method. CEC values of selected sorbents are shown in Table 2. Determination of initial cobalt concentration by GFAAS after sludge digestion confirmed the underline content of cobalt (<1 μg kg^−1^). It suggests the presence of unoccupied binding sites with free functional groups for sorption bivalent ions of heavy metals, i.e. Co^2+^ ions.

**Table 2:** Cation exchange capacity (CEC) and maximum sorption capacity (*Q*_max_) of selected sorbents for Co^2+^ ions

Sorbent	CEC (meq 100 g^−1^)	*Q*_max_ (μmol g^−1^)	Reference
Activated sludge from municipal WWTP	15	175 ± 17	This paper
Activated sludge from distillery WWTP	21	256 ± 9	[8]
Anaerobic sludge from paper mill WWTP	24	209 ± 5	[18]
Rhytidiadelphus squarrosus	-^a^	123 ± 6	[4]
Chlorella sp.	-^a^	246 ± 13	[19]
Bone char	-^a^	109 ± 5	[20]
Modified bentonite	51	2,343 ± 14	[21]

^a^CEC value was not studied

### Sorption characteristics of DAS for Co^2+^ ions

#### Optimization of sorption process

**Fig. 1:**
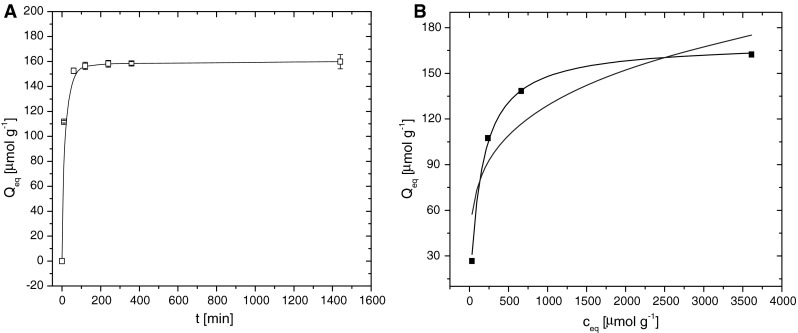
Effect of reaction time (5–1,440 min) on sortion process of Co^2+^ ions by DAS (2.5 g l^−1^) (**a**). Sorption conditions: *c*_0_ = 1,000 μmol l^−1^ CoCl_2_, A_0_ = 63 kBq l^−1 60^CoCl_2_, pH 6.0 ± 0.05, 22 °C. Models of Co^2+^ adsorption isotherms by DAS (**b**) (2.5 g l^−1^) under the terms of Langmuir (*solid line*) and Freundlich (*dot-dashed line*). Experimental DAS conditions: *c*_*0*_ = 1,000 μmol l^−1^ CoCl_2_, pH 6 ± 0.05, reaction time 4 h, 22 °C). All experiments were performed in triplicates. *Error bars* represent standard deviation of the mean (±SD)

Sorption process of cobalt ions by dried biomass of municipal activated sludge was rapid process and equilibrium was reached within 120 min (Fig. 1a). Increasing reaction time in the range from 240 to 1,440 min had non-significant effect to the uptake of Co^2+^ ions (*Q*_eq_) by DAS. After 24 h the *Q*_eq_ values calculated according Eq. 1 based on radiometric analyses of ^60^Co was 158 μmol Co g^−1^ (dw). Cobalt removing from single metal system reached 38 % from the total concentration of Co^2+^ ions in the reaction solution at time *t*_*0*_. Sorption process of heavy metals by microbial sorbents reached the reaction equilibrium within 2–6 h according to type of metal ion [22].

To assess the impact of CoCl_2_ initial concentration on the sorption capacity of sludge, we investigated the concentration range from 100 to 4,000 μmol l^−1^. Cobalt uptake by DAS increased with increasing initial concentration of Co^2+^ ions in solution (Fig. 1b). Saturation of DAS sorbent achieved at the initial concentration 1,000 μmol l^−1^ CoCl_2_, when sludge sorption capacity 154 μmol g^−1^ of Co^2+^ ions was reched. Van Hullebusch et al. [23] confirmed correlation between anaerobic sludge sorption capacity for cobalt ions and the initial concentration of Co^2+^ ions in the solution. The authors showed the level of sorbent saturation with maximum sorption capacity (*Q*_*max*_) 205 μmol g^−1^ CoCl_2_ at the time of reaction equilibrium. After time of equilibrium DAS sorption capacity has not changed in terms of occupied binding sites [8].

Mathematical models of sorption models offer parameters to identify, describe and understand the equilibrium of sorption processes [7, 24, 25]. Sorption isotherms can be used as useful tools for the determination and comparison of sorption capacities of various biomass types, as well as the affinity of metals to sorbent [26]. For describtion of obtained experimental data of Co^2+^ sorption equilibrium in single metal system Langmuir and Freundlich sorption isotherms were used (Table 1). Maximum sorption capacity of DAS for Co^2+^ ions obtained from Langmuir model was 175 ± 16 μmol g^−1 ^(dw). It confirmed the cooperation of wide range of sorbent surface binding sites in sorption process. From nonlinear regression analysis operation parameters of both models were obtained (Table 3). Equilibrium parameters and coefficients of determination indicated that Langmuir adsorption model fits the data better in comparison with Freundlich isotherm (Fig. 1b). The variance not explained by the model (100(1−*r* ^2^)) was lower than 3 % indicating that this adsorption model is adequate for description cobalt sorption behaviour by DAS. Isotherm coefficient *b* reveals the affinity of sorbent to Co^2+^ ions. Langmuir adsorption model shows more usable data for description the sorption process of Co^2+^ ions by anaerobic sludge compared to other mathematical models [23]. Evaluation of data by Freundlich model is suitable at lower concentrations of cobalt in reaction solution. Obtained value *Q*_max_ of sorbent prepared from dried municipal activated sludge for cobalt ions is comparable with sorption capacities of various biosorbents (Table 2). Differences between sorption capacities of sludges from municipal and industrial WWTP is notable. Our previous paper showed that dried distillery sludge contained higher concentration of unoccupied binding sites and thus its cation exchange capacity and maximum sorption capacity is superior.

**Table 3:** Model parameters sorption isotherms Co^2+^ ions dry mass of activated sludge component sorption system calculated by nonlinear regression

Type of sludge	Model	*Q*_*max*_ (μmol g^−1^)	*b* (dm^3^ μmol^−1^)	*K* (dm^3^ g^−1^)	*1/n*	*R* ^2^
DAS	Langmuir	174.5 ± 16.48	0.003 ± 0.001	–	–	0.9916
	Freundlich	–	–	21.91 ± 20.01	0.25 ± 0.13	0.7479

*R* ^2^—correlation coefficient

**Fig. 2:**
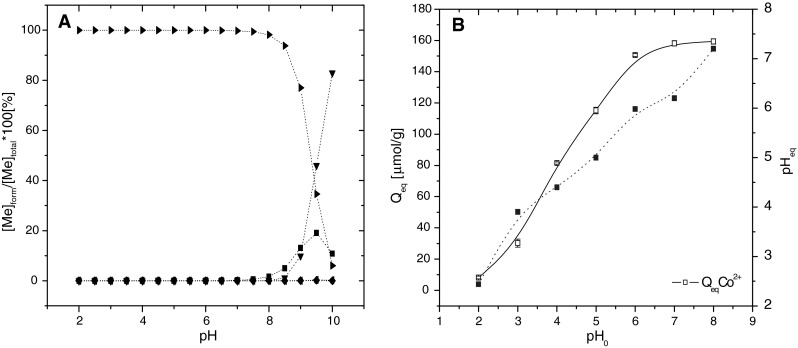
Cobalt speciation forms depending on the pH: Co^2+^ (), CoCl^−^ (), CoOH^+^ (), CoOH_2_ (), CoOH_3_^4−^ (). Used speciation program Visual MINTEQ ver. 2.53 under conditions: 1,000 μmol dm^3^ CoCl_2_, 22 °C, deionized water, p(CO_2_) 38.5 Pa (**a**). Effect of pH on sorption capacity of DAS for cobalt ions from the single component system at 22 °C, *c*_*0*_ 1,000 μmol/dm^3^ CoCl_2_, 31.3 g/dm^3^ biomass (dw) () and effect of initial pH on pH in equilibrium () (**b**)

Ionization equilibrium, speciation of metal and thus sorption process of metal ions by sewage sludge can be affected by pH value of reaction solution [7]. However characterisation of H^+^ ions impact to sorption capacity of sewage sludge is important to determine the form of cobalt presented in the sorption system at the studied pH. Results of cobalt speciation analyses by Visual Minteq confirmed Co^2+^ as the main form (>99.5 %) of cobalt in aqueous solution with initial concentration 1,000 μmol dm^−3^ of CoCl_2_ in the pH range 2–8 (Fig. 2a). At pH > 9 uptake of cobalt ions by DAS decresed corresponding with the formation of unsoluble cobalt hydroxide form Co(OH)_2_ and Co (OH)^+^ and its precipitation on sorbent surface. In strong acidic reaction solution, H^+^ ions compete for active sorption sites with cobalt ions and reduce effectivity of metal removal. The concentration of Co^2+^ ions sorbed by DAS decreased with acidity increasing [8]. Figure 2a also shows the effect of initial pH values on terminal pH values of reaction system. The terminal pH marks value in time of sorption equilibrium. Microbial biomass of sewage sludge and produced extracellular polymers are sensitive and can be affected by pH of reaction solution [27]. Generally, sorption and accumulation process of heavy metals is a function of pH for fungi [28], algae [29], moss [4] or bacteria [2]. All of authors show the crucial role of ion exchange in sorption process. Sasso et al. [30] investigated the maximal sorption capacity of sewage sludge for lead and cadmium ions in the range 2–5.

#### The mechanism of Co^2+^ ions sorption process by DAS—role of functional groups of sludge surfaces

**Fig. 3:**
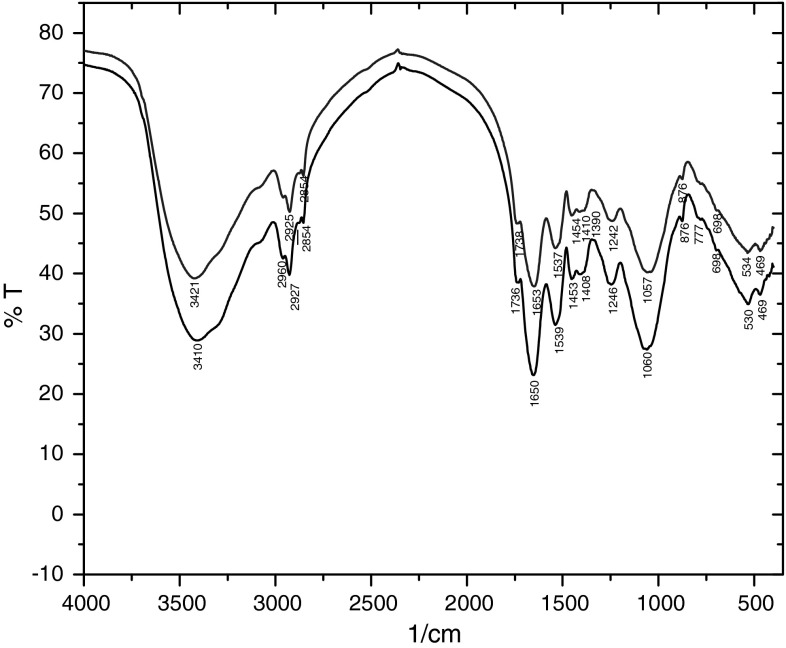
FT-IR spectrum of DAS before (–) and after (–) sorption proceses of Co^2+^ ions (*c*_0_ = 1,000 μmol dm^3^ CoCl_2_, DAS 2.5 g dm^3^, dw)

The FT-IR spectra of DAS before and after cobalt sorption are shown in Fig. 3 and the spectra are summarized in Table 4. Spectrum of sludge is complicated, reflecting the complex nature of the dried biomass. Despite this complexity some characteristic peaks can be assigned. Peak size reflects the relative concentration of functional groups [9]. The position and shape of adsorption peak at 3,410 cm^−1^ before cobalt sorption represents self-associated OH groups.

The peaks at 2,960 and 2,854 cm^−1^ are due to C–H stretching vibration of CH, CH_2_ and CH_3_ groups. The broad and strong bands at 1,650 and 1,539 were attributed to asymmetric and symmetric stretching of C=O and C–N, respectively. The intense peak at 1,040 cm^−1^ could be assigned to O–H groups. These assignments are consistent with the presence of carboxylic and amino groups. These groups are characteristic of bacterial cell wall of bacteria that generally coexist in biological plants for water treatment [13].

An analysis of the FT-IR spectrum after Co^2+^ ions sorption showed that there were substantial changes in the adsorption intensity of amide groups at 1,539 and 1,635 cm^−1^ and carboxylates at 1,736 and 1,246 cm^−1^. This indicates that amide and carboxylate groups of DAS play a crucial role in binding of Co^2+^ ions. Hydroxyl groups are also involved in cobalt binding process to some degree. This finding is consistent with that supported by our previous study [8] for Co^2+^ ions by dried activated sludge from industrial WWTP. Carboxyl and hydroxyl groups are the main binding sites for Co^2+^ sorption by aerobic sludge granules [31] (Table 4).

**Table 4:** The values of wavenumber (cm^−1)^ of DAS and corresponding functional groups

Assignment	ν cm^−1^	
	DAS	
	Band before Co^2+^ sorption	Band after Co^2+^ sorption
O–H stretching vibration	3,410	3,423
C–H stretching (asymmetric) vibration	2,960	2,956
C–H stretching (symmetric) vibration	2,854	2,852
C=O stretching vibration	1,736	1,736
C=O and C–N stretching vibration (amide I)	1,650	1,657
C–N stretching vibration (amide II)	1,539	1,518
O–H stretching vibration	1,408	1,427
=C–O–C stretching vibration	1,246	1,230
O–H stretching vibration (primary OH)	1,060	1,078

**Fig. 4:**
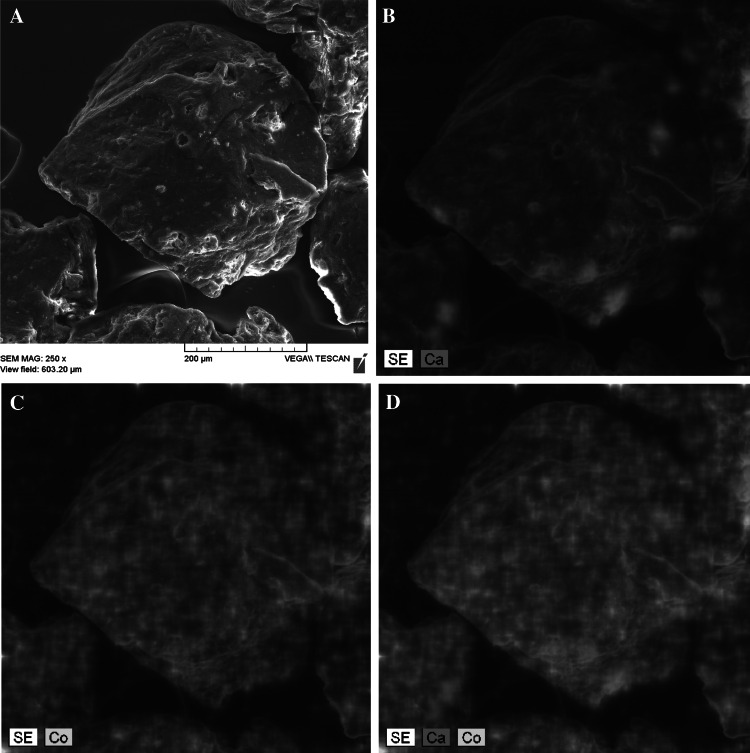
SEM pictures of Ca-treated DAS after Co^2+^ ions biosorption (4,000 μmol dm^3^ CoCl_2_) at pH 6.0; magnification ×250 (**a**). EDX element mapping of Ca (**b**), element mapping of Co (**c**) and element mapping of Ca and Co on DAS surface (**d**)

SEM connected with EDX is a powerful technique that can be used to investigate binding of metals to sorbent [7, 32]. This method enables to monitor morphological changes in the surface after sorption and also provides valuable informations about distribution of various elements on sorbent surface. In this paper SEM-EDX analysis was carried out to characterize DAS surface before and after Co^2+^ ions uptake in single system. Comparison of sorbent SEM images before and after Co^2+^ sorption shows that there are no morphological changes on the biomass surface as well as that there is no specific localization of cobalt on sorbent surface (data not shown). Confirming that ion-exchange participates in Co biosorption required treating of sorbent with CaCl_2_. Figure 4 clearly demonstrates distribution of Ca on Ca-treated biosorbent surface. Ca distribution seems to be non-uniform except for areas with intense red color indicating the precipitation of Ca in the form of CaCO_3_. Figure 4c depict SEM images of DAS surface after cobalt biosorption. As can be seen adsorbed cobalt was uniformly distributed on DAS surface apart from areas where calcium ions precipitated. This suggests that negatively charged functional groups have exchanged Ca^2+^ ions with Co^2+^ ions from aqueous solution and this implies that ion-exchange participates in cobalt sorption. However, it is evident (Fig. 4d) that there was no ion-exchange observed on places where Ca precipitated.

## Conclusions

Huge amounts of sewage sludges produced by WWTPs represent serious environmental problem of increased agglomerations and industrial intensification. Sludge with low concentrations of toxic metals and other xenobiotics can be utilized as agricultural fertilizers and soil conditioners. One of the sludge utilizations is application this removable biomaterials in processes of toxic metals sorptions from diluted liquid wastes and thus concentrate its self. Application of suitable radioisotope can be effective to monitoring and description toxic metal mobility. In our paper radio indicator method by radionuclide ^60^Co and γ-spectrometry for characterization DAS sorption properties was used. Data presented in our paper confirm high sorption capacity of dried activated sludge from municipal WWTP for Co^2+^ ions up to 175 ± 9 μmol g^−1^. Sorption capacity is pH dependent and increasing in the interval from 2 to 8. FT-IR spectroscopy confirmed the main binding sites in cobalt sorption as carboxyl, hydroxyl and amino groups. SEM-EDX analysis confirmed ion-exchange mechanism of Co^2+^ ions by DAS. The main disadvantage of DAS sorbent is partial solubilization of proteins and thus change in reactivity with chemical agents.
